# Complete Genome Sequences of Two *Escherichia coli* Phages, vB_EcoM_ ESCO5 and vB_EcoM_ESCO13, Which Are Related to phAPEC8

**DOI:** 10.1128/genomeA.01337-16

**Published:** 2017-03-30

**Authors:** Angélina Trotereau, Mathieu Gonnet, Antoine Viardot, Anne-Christine Lalmanach, Rodrigo Guabiraba, Nathalie Katy Chanteloup, Catherine Schouler

**Affiliations:** ISP, INRA, Université François Rabelais de Tours, Nouzilly, France

## Abstract

We report here the complete genome sequences of two *Myoviridae* phages that infect various avian-pathogenic *Escherichia coli* strains and that are closely related to phage phAPEC8.

## GENOME ANNOUNCEMENT

Phages are promising biocontrol agents for avian *Escherichia coli* infections (1–3). Coliphages vB_EcoM_ESCO5 and vB_EcoM_ESCO13 were isolated in France in 2015 from the cecal contents of a ROSS PM3 chicken and a water sample from a sewage treatment plant, respectively. They were isolated by propagation on the avian-pathogenic *E. coli* strains BEN5202 of serogroup O2:K1 and BEN4311 of serogroup O1:K1, respectively. They both exhibited a broad host range, since they were able to propagate on *E. coli* strains of various serogroups (O1, O2, O78, O88, O6, O8, O18, and O25), i.e., 32 and 27 out of 46 tested strains for ESCO5 and ESCO13, respectively. Negative staining of phages and transmission electron microscopic analysis showed that ESCO5 and ESCO13 belong to the *Myoviridae* family. One-step growth experiments were performed to determine the kinetic parameters on strain BEN3801 O18:K1 (4). Both phages had a latent phase of 15 min and a rise phase of 40 min. ESCO5 had a burst size of 135 and ESCO13 had a burst size of 42. Unlike ESCO5, ESCO13 is less able to adsorb well on BEN3801 (adsorption constants of 3.00 10^-9^ ml/min and 4.78 10^-10^ ml/min, respectively) and do not induce total lysis of the bacteria.

DNA phage libraries were prepared with the Nextera kit (Illumina) and sequenced to 2 × 250 read length on a MiSeq system (Illumina) by the DNA Sequencing Facility of the University of Cambridge (UK). Reads were trimmed with Sickle and assembled with SPAdes 3.1.1 (5). Coding sequences (CDSs) were predicted and annotated using Prokka (6, 7). Gene products were controlled by protein similarity search using BlastP (UniProt) (8).

ESCO5 and ESCO13 are DNA double-stranded phages with genome sizes of 149,312 bp and 149,813 bp, and their G+C contents are 39.1% and 38.9%, respectively. They are highly homologous to phage vB_EcoM_phAPEC8 (9). Two hundred seventy-five genes and 291 genes were predicted in the genomes of ESCO5 and ESCO13, respectively. They shared 249 genes with phage phAPEC8. Six, 10, and eight genes are specific for ESCO5, ESCO13, and phAPEC8, respectively, mostly of unknown function. Phages phAPEC8 and ESCO5 have in common a gene coding for endo-*N*-acetylneuraminidase, a gene that is not present in ESCO13. The endo-*N*-acetylneuraminidase is 96.7% identical to the endosialidase EndoN92, encoded by phage phi92 (10). EndoN92 is present on the phage particle and digests polysialic acid of the K1 capsule (11). ESCO5 and ESCO13 are not K1-dependent phages, since they are able to propagate on strains that are K1 negative (K1-neg), such as O78 and O88 strains. Thus, the K1 capsule is not their receptor, and the endosialidase probably allows ESCO5 to better reach its receptor when infecting *E. coli* K1 than ESCO13.

No gene related to lysogeny, such as integrases or specific recombinases, was identified in the genomes of ESCO5 and ESCO13. Moreover, another phAPEC8-related phage was identified by metagenomics analysis from a commercial Russian phage cocktail which is constituted of at least 10 different phage genera (12). These data combined with broad host range of ESCO5 and ESCO13 reinforce their potential use as therapeutic agents.

### Accession number(s).

The complete genome sequences of these two phages have been deposited in GenBank under the accession numbers KX664695 (ESCO5) and KX552041 (ESCO13).
